# Sub-second pair distribution function using a broad bandwidth monochromator

**DOI:** 10.1107/S1600576723004016

**Published:** 2023-05-31

**Authors:** Nicolas P. L. Magnard, Daniel R. Sørensen, Innokenty Kantor, Kirsten M. Ø. Jensen, Mads R. V. Jørgensen

**Affiliations:** a Department of Chemistry and Nano-Science Center, University of Copenhagen, Copenhagen 2100, Denmark; b Department of Chemistry & iNANO, Aarhus University, Aarhus 8000, Denmark; c MAX IV Laboratory, Lund University, Lund 224 84, Sweden; d Department of Physics, Technical University of Denmark, Lyngby 2880, Denmark; Australian Synchrotron, ANSTO, Australia

**Keywords:** total scattering, pair distribution function, metal oxo clusters, synchrotron radiation

## Abstract

Here the use of a broad energy bandwidth monochromator, *i.e.* a pair of B_4_C/W multilayer mirrors (MLMs), is demonstrated for X-ray total scattering (TS) measurements and pair distribution function (PDF) analysis. Data are collected both on powder samples and from metal oxo clusters in aqueous solution at various concentrations. A comparison between the MLM PDFs and those obtained using a standard Si(111) double-crystal monochromator shows that the measurements yield MLM PDFs of high quality which are suitable for structure refinement. Moreover, the effects of time resolution and concentration on the quality of the resulting PDFs of the metal oxo clusters are investigated. PDFs of heptamolybdate clusters and tungsten α-Keggin clusters from X-ray TS data were obtained with a time resolution down to 3 ms and still showed a similar level of Fourier ripples to PDFs obtained from 1 s measurements. This type of measurement could thus open up faster time-resolved TS and PDF studies.

## Abstract 2

This work demonstrates the use of a broad energy bandwidth multilayer mirror monochromator for X-ray total scattering (TS) measurements and pair distribution function (PDF) analysis. This type of measurement could facilitate faster time-resolved TS and PDF studies.

## 1. Introduction

Total scattering (TS) and pair distribution function (PDF) analysis have become important techniques for structural characterization of amorphous compounds, nanoparticles and nanoclusters where conventional structural methods (*i.e.* diffraction) fail due to the small coherence length scales and atomic disorder (Gulenko *et al.*, 2014 ▸; Narten *et al.*, 1982 ▸; Liang *et al.*, 2014 ▸; Ding *et al.*, 2015 ▸; Masadeh *et al.*, 2007 ▸; Yang *et al.*, 2013 ▸; Andersen *et al.*, 2021 ▸; Christiansen *et al.*, 2020 ▸). The TS technique relies on measuring both the Bragg and the diffuse scattering to high momentum transfers, *Q*, often up to and above 15 Å^−1^ for *in situ* experiments and even higher for *ex situ* measurements (Billinge, 2004 ▸). The scattering data are subsequently used to calculate the PDF, a real space function, which is analyzed. Extraction of an accurate PDF is contingent on a high signal-to-noise ratio in the whole *Q* range used.

The requirement for high-quality data at high *Q* values has in the past been difficult to achieve for X-rays due to the rapid decrease of the atomic form factor leading to weak scattering at high *Q*. However, with the emergence of third, and now fourth, generation synchrotron storage rings, TS experiments have been made available to a larger community (Billinge, 2019 ▸). Using these high-flux X-ray sources, in combination with large and effective 2D area detectors, it is now possible to measure X-ray TS patterns with a time resolution of approximately 1 s, even on poorly scattering systems (Chupas *et al.*, 2003 ▸; Beauvais *et al.*, 2022 ▸). This has enabled *in situ* PDF experiments (Aalling-Frederiksen *et al.*, 2021 ▸; Anker *et al.*, 2021 ▸; Grote *et al.*, 2021 ▸); however, experiments with faster time resolution, or data collection from samples in low concentration, are often impossible due to a low signal-to-noise ratio at high *Q*. In other words, many TS experiments are flux limited and necessarily designed as a compromise between time and real space resolution (Roelsgaard *et al.*, 2023 ▸).

The primary monochromator used at most diffraction and scattering beamlines is a double-crystal monochromator (DCM). The energy bandwidth of these depends on the type of crystal (Si, diamond, Ge,…) and the reflection (111, 311, 511,…) used but is most often on the order of Δ*E*/*E* ≃ 10^−4^ or smaller. This bandwidth is well suited for medium- to high-resolution diffraction experiments; however, it only transmits a small fraction of the photons in a white/pink synchrotron beam. Multilayer mirrors (MLMs), made with alternating layers of light and heavy material, have reflectivity profiles with peak reflectivity which often exceeds 90% and bandwidths of ∼0.2–2%. Utilizing a set of these MLMs, it is possible to build a monochromator with a much wider energy bandwidth than a standard DCM. The multilayers can be designed to match the bandwidth of the harmonics from the undulator insertion device commonly found as the X-ray source at synchrotrons, thus increasing the flux on samples by up to two orders of magnitude compared with a conventional Si(111) monochromator (De Nolf *et al.*, 2009 ▸) at the cost of a proportional energy resolution loss.

Here we investigate the feasibility of using an MLM monochromator for TS experiments. We perform TS experiments on metal oxo clusters in solution by varying several parameters, namely the *Z* of the metal ion in the cluster, the concentration of clusters in solution and the acquisition rate of the scattering pattern. Metal oxo clusters are large ions that form in aqueous solutions constituted by one or more metal atoms, coordinated to O^2−^, HO^−^ or H_2_O (Coronado & Gómez-García, 1998 ▸; Anker *et al.*, 2021 ▸). These clusters are usually found in solutions of Mo, W, Nd, Zr or Hf (Gumerova & Rompel, 2018 ▸, 2020 ▸), but recent studies have shown metal oxo clusters formed by a larger range of transition metals (Sadeghi *et al.*, 2015 ▸; Zhang *et al.*, 2013 ▸; Zheng & Yang, 2012 ▸; Goberna–Ferrón *et al.*, 2015 ▸). PDF analyses of oxo clusters have been reported in the literature; however, high concentrations and/or long exposure times and/or limited *Q* range have been employed to reliably access usable PDFs from these weakly scattering systems (Anker *et al.*, 2021 ▸; Juelsholt *et al.*, 2019 ▸; Nielsen *et al.*, 2021 ▸). We show that, although the same real space resolution as with a classic Si(111) DCM can be achieved, the time resolution can be increased by at least an order of magnitude, enabling the acquisition of PDF data with sub-second time resolution while maintaining high real space resolution.

## 2. Methods

### 2.1. Sample preparation

All chemicals used were supplied by Sigma–Aldrich, except ammonium metatungstate hydrate supplied by Alfa Aesar. Aqueous solutions of metatungstate [W_12_O_40_]^6−^, hepta­molyb­date [Mo_7_O_24_]^6−^, calcium–manganese [Ca_2_Mn_3_O_20_]^6−^ and strontium–manganese [Sr_2_Mn_3_O_20_]^6−^ clusters were prepared. For the metatungstate and heptamolybdate cluster solutions, a stock solution with a concentration of 0.50 *M* was first prepared by dissolving 3 mmol of ammonium metatungstate hydrate [(NH_4_)_6_W_12_O_39_·*x*H_2_O] and ammonium heptamolybdate tetrahydrate [(NH_4_)_6_Mo_7_O_24_·4H_2_O] in 6 ml of deionized (DI) water. A concentration series from the metatungstate cluster stock solution was prepared with concentrations of 0.10, 0.05 and 0.01 *M*. For the 0.10 *M* solution, 1 ml of the stock solution was diluted in 4 ml of DI water; for 0.05 *M*, 1 ml of stock solution was diluted in 9 ml of DI water; and for 0.01 *M*, 0.1 ml of stock solution was diluted in 4.9 ml of DI water. The same procedure was applied for the heptamolybdate cluster concentration series.

For the calcium–manganese and strontium–manganese cluster solutions, 6 mmol of manganese chloride tetrahydrate (MnCl_2_·4H_2_O) and calcium chloride dihydrate (CaCl_2_·2H_2_O) or strontium chloride hexahydrate (SrCl_2_·6H_2_O) were dissolved together in 6 ml of DI water in order to reach [Mn^2+^] and [Ca^2+^/Sr^2+^] concentrations of 1.00 *M*. A volume of 3 mmol of ammonium persulfate [(NH_4_)_2_S_2_O_8_] was added to the stock solutions and stirred until a brown solution appeared. The stock solutions were then diluted to 0.50 and 0.10 *M* solutions by mixing 2 ml of the stock solution in 2 ml of DI water and 1 ml of the stock solution with 9 ml of DI water, respectively. All solutions of the four cluster systems were injected into 1.0 mm borosilicate glass capillaries and sealed with ep­oxy resin.

### 2.2. X-ray total scattering

X-ray TS data on the polyoxometalate solutions as well as on the DI water and 0.5 *M* ammonium persulfate solutions were collected at the DanMAX beamline at the MAX IV Laboratory (Lund, Sweden). Data were also collected from LaB_6_ (NIST SRM660c) and Si (NIST SRM640f) standards, loaded into 0.2 and 1.0 mm borosilicate capillaries, respectively. The capillaries were placed and aligned on a capillary spinner with a horizontal rotation axis to spin the samples during the measurements to prevent potential sedimentation. TS data were acquired first using an Si(111) DCM and subsequently using a B_4_C/W MLM. The photon energy used for both monochromators was 35 keV. All measurements were performed at 300 mA beam current. During the experiment, vacuum issues in the MLM required that the beam acceptance was reduced, and thus the resulting flux is approximately half of the current maximum possible flux at DanMAX. The flux of the DCM and MLM beams was measured using a photodiode to ∼4.0 × 10^11^ and ∼2.0 × 10^13^ photons s^−1^, respectively. Moreover, their respective bandwidths are Δ*E*/*E* ≃ 1.4 × 10^−4^ and Δ*E*/*E* ≃ 7.7 × 10^−3^. The scattering signal was collected using a DECTRIS PILATUS3 X CdTe 2M area detector, with the direct beam impinging near the center of the bottom side of the detector. The beam cross section used for this study was square shaped with the dimensions 0.8 × 0.8 mm. The sample-to-detector distance was 100 mm. In this geometry, the parallax effect can cause a peak shift at high *Q*. This is expected and can be corrected (Marlton *et al.*, 2019 ▸). However, because the detector employed in this study uses a CdTe detection layer, photons with a high incidence angle are absorbed up to 99.95% within 0.1 mm, *i.e.* 0.5 pixels. Integrated patterns with and without corrections were essentially identical.

The 2D detector images were integrated using *pyFAI* (Kieffer *et al.*, 2020 ▸) through the *Dioptas* program (Prescher & Prakapenka, 2015 ▸) and PDFs were obtained using the *PDFgetX3* package (Juhás *et al.*, 2013 ▸). *Q*_max instrument_ and *Q*_max_ values of 19.5 Å^−1^ could be achieved, *Q*_min_ = 0.7 Å^−1^, and the *r*_poly_ value was set to 1.1. PDF refinements were performed using *TOPAS* (Coelho, 2018 ▸).

## 3. Results and discussion

### 3.1. Performance and instrumental resolution of DCM versus MLM

**Figure 1:**
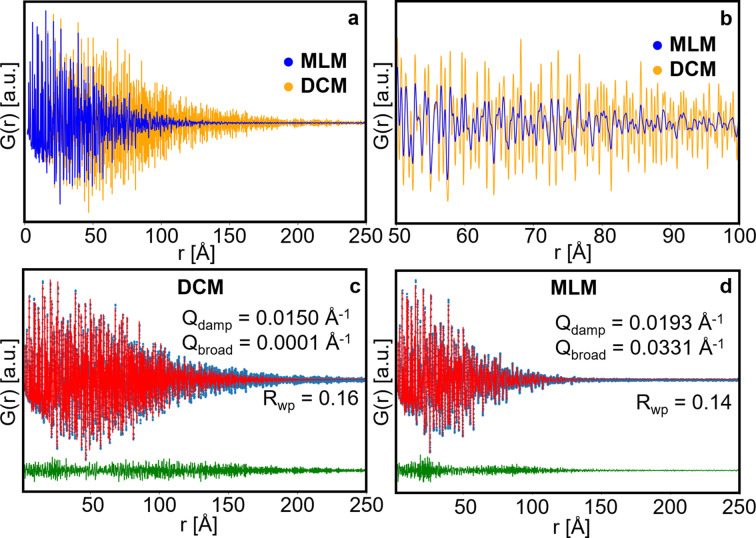
(*a*) Comparison of the PDF obtained from LaB_6_ standard powder samples in a Ø0.2 mm glass capillary using the DCM (orange curve) and the MLM (blue curve). (*b*) Enlargement of the PDF range 50–100 Å. (*c*) Refinement of the LaB_6_ structure (*Pm* 3 *m*) to the DCM PDF, where *Q*_damp_ and *Q*_broad_ are refined. (*d*) Refinement of the LaB_6_ structure (*Pm* 3 *m*) to the MLM PDF, where *Q*_damp_ and *Q*_broad_ are refined. In (*c*) and (*d*), the experimental PDF is shown in blue, the calculated PDF in red and the difference between them in green.

X-ray TS patterns were first acquired on an LaB_6_ standard powder sample in a Ø0.2 mm borosilicate capillary using both the DCM and MLM. The corresponding PDFs are shown in Fig. 1 ▸(*a*), where a large difference in the instrumental damping is observed. In the DCM PDF, peaks up to 170–180 Å can be observed, while the PDF signal from the MLM disappears around 120 Å. The difference observed is however caused not only by damping but also by the *r*-dependent broadening of the peaks in the MLM PDF [see Fig. 1 ▸(*b*)].

Both the experimental PDFs can be described by the LaB_6_ structure (space group *Pm* 3 *m*). *Q*-space (PXRD) and real space (PDF) Rietveld refinements against the two datasets are shown in Fig. S1 of the supporting information and Figs. 1 ▸(*c*)–1 ▸(*d*), respectively. Here, the cell parameter, the *x* fractional coordinate of the B atoms, and isotropic atomic displacement parameters (ADPs) of La and B were refined. The refinement results are given in Table S1 of the supporting information. In the case of the *Q*-space Rietveld refinement, a TCHZ peak shape is used, where the *U*, *V*, *W* and *Y* parameters are refined. To investigate the effect of damping of the PDF signal by the broad energy distribution of the MLM, the *Q*_damp_ and *Q*_broad_ parameters are refined. These two parameters account for the impact of the instrumental resolution of the instrument on the experimental PDF. The first parameter quantifies the damping of the PDF at high *r*, and the latter quantifies the *r*-dependent broadening of the PDF peaks. It is noticeable that the *Q*_broad_ parameter is mostly impacted, increasing from 0.0001 (3) Å^−1^ for the DCM to 0.0415 (4) Å^−1^ for the MLM data. The increase of *Q*_damp_ is significant (26%), increasing from 0.0149 (1) to 0.0187 (5) Å^−1^ for the DCM and MLM data, respectively. However, the majority of of the difference in the PDFs can be explained by *Q*_broad_. Furthermore, the refined values of the La and B ADPs for the MLM PDF data are higher than those of the DCM. This could be related to the larger energy bandwidth of the MLM which gives rise to broader features in *Q* and *r* space, making it more difficult to estimate the effects of the sample on the PDF. We will examine such effects below.

TS data were also collected from a silicon powder sample in a Ø1.0 mm borosilicate capillary. The DCM and MLM PDFs including refinements, as well as *Q* space Rietveld refinements, are shown in Fig. S2 and the refined parameters are summarized in Table S2. We note the high amount of noise in the DCM PDF of the Si standard, which can be observed in the difference curve of its refinement [see Fig. S2(*e*)]. Nevertheless, the difference in the damping effect observed with the MLM data compared with the DCM data is less pronounced when using larger sample diameters [see Figs. S2(*c*)–S2(*d*)], indicating that there is a predominant effect of sample size over the energy bandwidth on the damping parameter of the Bragg peak broadening. As expected, *Q*_broad_ is higher for the MLM PDF than the DCM PDF.

**Figure 2:**
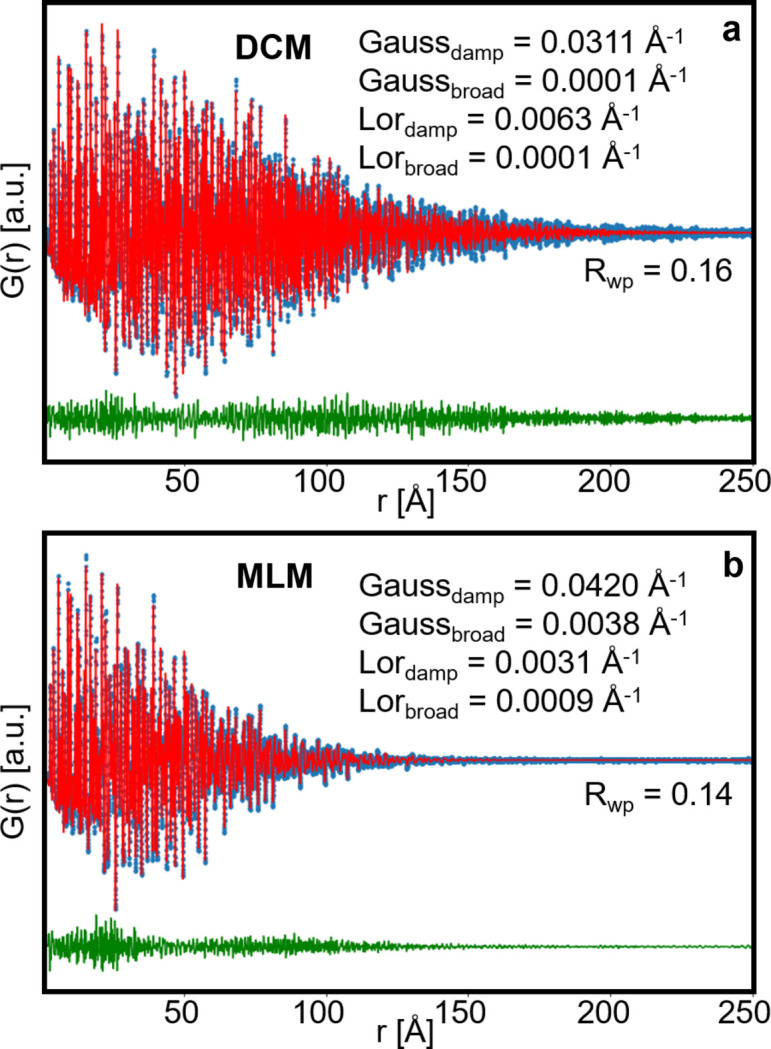
(*a*) Refinement of the LaB_6_ structure (*Pm* 3 *m*) on the DCM PDF using a Voigt profile as described by Beyer *et al.* (2022 ▸). (*b*) Refinement of the LaB_6_ structure (*Pm* 3 *m*) on the MLM PDF using the same type of Voigt profile. The experimental PDF is shown in blue, the calculated PDF in red and the difference between them in green.

To further characterize the difference in PDF peak profile between the DCM and MLM, we apply the analysis recently proposed by Beyer *et al.* (2022 ▸). Here the individual Gaussian and Lorentzian contributions of a Voigt profile in *Q* space are used to describe the PDF in real space. In total, four parameters are refined for the instrumental broadening: Gaussian *Q*_damp_ and *Q*_broad_, and Lorentzian *Q*_damp_ and *Q*_broad_. The results of the PDF refinements against the LaB_6_ PDFs using this approach are shown in Fig. 2 ▸ with the refinement results shown in Table S3. Compared with the simpler model described above, this one provides more thorough information on the instrumental effect on the broadening of peaks in *Q* space and in real space, even if it does not improve the fit. In both cases the dominating terms are the damping, especially the Gaussian contribution. The Lorentzian contribution is significant for both, but higher for the DCM PDF compared with the MLM PDF. Both the broadening terms are negligible for the DCM PDF. For the MLM PDF, the Gaussian broadening term dominates, but a significant fraction of the broadening can be described with the Lorentzian term. This effect of the MLM on the broadening of PDF peaks can be related to the mathematical description of a Voigt profile in *Q* space. The Gaussian and Lorentzian components of the Voigt profile width can be separated into two terms each, *Q*-independent terms *K*_G_ and *K*_L_, and *Q*-dependent terms δ_G_ and δ_L_. The *Q*-independent terms lead to damping of the PDF, whereas the *Q*-dependent terms impact the broadening of the PDF peaks (Beyer *et al.*, 2022 ▸). Surprisingly, although the simple model used above indicated a *Q*_broad_ contribution of the same order of magnitude as *Q*_damp_ in the MLM data, the former is this time an order of magnitude smaller than the latter in the advanced model.

When examining the correlation matrix of the refined parameters of both types of refinements, namely the simple *Q*_damp_ and *Q*_broad_ model and the one including Gaussian and Lorentzian contributions, correlations were observed between the different refined parameters. In the case of the simple model, the refined parameters are correlated in the same way in both the DCM PDF and the MLM PDF [Figs. S3(*a*)–S3(*b*)], especially *Q*_damp_, *Q*_broad_, the La ADP and the scaling factor. *Q*_broad_ shows an anti-correlation with the La ADP. This makes sense as higher broadening of the PDF peaks will appear similar to increased thermal motion. Moreover, these parameters are inherently correlated considering the way in which they are implemented in *PDFgui* and in *TOPAS* (Neder & Proffen, 2020 ▸). The *Q*_damp_ and La ADP parameters are positively correlated with the scaling factor. This can be explained as the latter compensating for the decrease in intensity of the PDF peaks as *Q*_broad_ and/or the La ADP increase. The same type of interpretation can be achieved on the negative correlation of *Q*_damp_ with the scaling parameters, since the increase of *Q*_damp_ (*i.e.* a more rapid damping of the PDF at high *r*) hinders a large scale factor. In the case of the approach of Beyer *et al.* (2022 ▸) [Figs. S3(*c*)–S3(*d*)], a strong anti-correlation of the Gaussian broadening factor arises with several parameters for the MLM data, notably the Gaussian damping, Lorentzian broadening and scale factors, and the La ADP. In the case of the DCM PDF, both Gaussian and Lorentzian broadening terms are insignificant, and the correlation is thus meaningless. In contrast to the simple model, the approach from Beyer *et al.* (2022 ▸) ensures that the broadening terms are not intrinsically correlated with the ADPs. The stronger (anti-)correlation in the MLM model could arise from the broadening of peaks in the MLM data that makes it harder to deconvolute instrumental effects from the sample parameters. To illustrate this, we have modeled the Bragg peaks of the LaB_6_ standard measured with MLM and DCM [Fig. S4(*a*)] using a Voigt profile and extracted the half-width at half-maximum (HWHM) of each peak. These data are plotted against the momentum transfer *Q*, shown in Fig. S4(*b*). By fitting a linear regression on both curves, we notice that the peak broadening in the DCM data is largely independent of *Q*, whereas the MLM data have a strong *Q* dependence, corroborating the observations discussed above in this section. To take a step further in the quantification of the instrumental resolution, an instrumental resolution function (IRF) for both the DCM and the MLM is calculated according to the Caglioti approach. Here the full width at half-maximum (FWHM) of diffraction peaks follows the relation (FWHM)^2^ = *U*tanθ^2^ + *V*tanθ + *W*. The resulting IRF is shown in Fig. S4(*c*). Similarly to the linear fitting of HWHM as a function of *Q*, one can notice that the MLM IRF displays a strong *Q* dependency of the FWHM. The energy spectrum of the MLM X-ray beam has a nearly Gaussian distribution [see Fig. S4(*d*)], which can explain the stronger Gaussian behavior of the damping and broadening terms of the MLM PDF. This impact of a large bandwidth on the PDF has been emphasized to a greater extent by Soper & Barney, (2011 ▸) with the use of the white beam of an Ag source to measure TS.

### 3.2. MLM-PDF on metal oxo clusters

**Figure 3:**
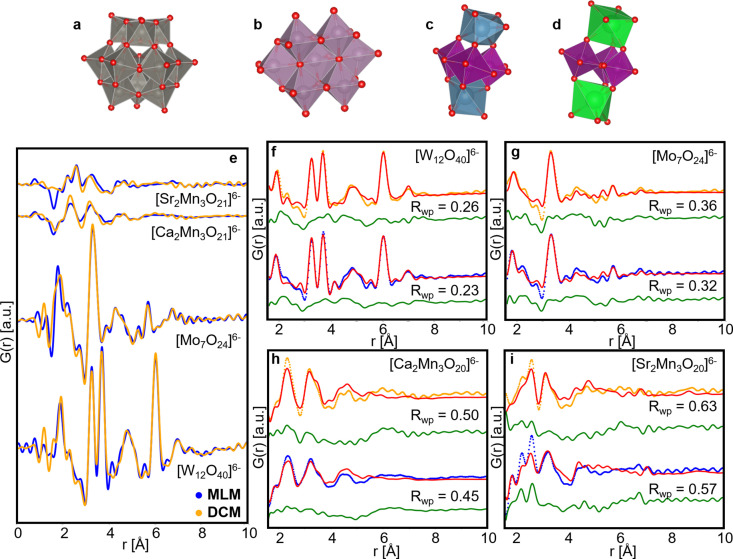
Structures of the (*a*) [W_12_O_40_]^6−^ α-Keggin ion, (*b*) [Mo_7_O_24_]^6−^ cluster, (*c*) [Ca_2_Mn_3_O_20_]^6−^ cluster and (*d*) [Sr_2_Mn_3_O_20_]^6−^ cluster. (*e*) Comparison of the PDF from [W_12_O_40_]^6−^, [Mo_7_O_24_]^6−^, [Ca_2_Mn_3_O_20_]^6−^ and [Sr_2_Mn_3_O_20_]^6−^ cluster solutions at the highest concentrations acquired with the DCM (orange curves) and MLM (blue curves). (*f*) Refinement of the α-Keggin structure to the [W_12_O_40_]^6−^ data, (*g*) heptamolybdate structure to the [Mo_7_O_24_]^6−^ data, (*h*) a calcium–manganese cluster structure previously reported by Nayak *et al.* (2011 ▸) to the [Ca_2_Mn_3_O_20_]^6−^ data and (*i*) a strontium–manganese structure adapted from Nayak *et al.* (2011 ▸) to the [Sr_2_Mn_3_O_20_]^6−^ data. In (*f*)–(*i*) the experimental MLM PDF is shown in blue, while the experimental DCM is shown in orange. The calculated PDF from the model structures is shown in red and the difference curve in green.

Having shown that the PDFs obtained with the MLM can be used for structure refinements, albeit yielding a lower resolution limit in real space, we now turn our focus to the metal oxo clusters. TS data were collected on metal oxo cluster solutions with 1 s exposure from the samples with the highest cluster concentration ([W_12_O_40_]^6−^, [Mo_7_O_24_]^6−^: 0.50 *M*; [Ca_2_Mn_3_O_20_]^6−^, [Sr_2_Mn_3_O_20_]^6−^: 1.00 *M*). Due to the higher amount of noise at high *Q*, *Q*_max_ has been fixed to 18.0 Å^−1^ to calculate the PDF of the samples studied in this section. For the tungsten and molybdenum solutions, the scattering pattern of DI water has been scaled and subtracted from each scattering pattern in the background subtraction process. For the manganese solutions, the scattering pattern of the 0.5 *M* ammonium persulfate solution has been scaled and subtracted from each scattering pattern. The resulting PDFs are shown in Fig. 3 ▸(*e*). PDFs obtained with the DCM are shown in orange and with the MLM are shown in blue. The PDFs show the same peaks, albeit with some variations in the high-frequency Fourier ripples below 1.5 Å and above 6 Å originating from high-*Q* noise, where PDF peaks are not observed or expected.

Metatungstate cluster solutions are well known to contain α-Keggin ions ([W_12_O_40_]^6−^) (Juelsholt *et al.*, 2019 ▸), while ammonium heptamolybdate dissolves to [Mo_7_O_24_]^6−^ clusters. The structure of the manganese cluster found here has been reported by Nayak *et al.* (2011 ▸). The structure of the clusters has been extracted from CIFs from the ICSD databank: No. 71173 for the [W_12_O_40_]^6−^ cluster, No. 4153 for the [Mo_7_O_24_]^6−^ cluster and No. 7015300 for the manganese clusters. In the case of the strontium–manganese clusters, the two *A* ^2+^ sites that are formerly Ca^2+^ in the publication have been substituted by Sr^2+^. Using these as starting models and refining against the experimental PDFs [Figs. 3 ▸(*f*)–3(*i*)] we see that these structures describe the features in the PDF well. Initially, cell parameters, ADPs, scale and the Delta2 parameter were refined, and in a second refinement cycle the metal site atomic positions were refined. Refinement details are given in Tables S4–S7. The fits to the MLM PDFs generally yield lower *R*_wp_ values compared with the DCM PDFs. This is probably caused by the higher flux from the MLM, yielding an improved signal-to-noise ratio. On the basis of these results we confirm that TS and subsequent PDF analysis using a broad bandwidth beam is feasible also for nanostructured samples. Further, we observed that the results are on par with, or superior to, those obtained with a regular DCM. The PDF refinement of the manganese clusters have a higher *R*_wp_ value than those of the tungsten and molybdenum clusters, and the model does not describe all features in the data. The structural model used for these refinements is extracted from a structure refined on single-crystal data (Nayak *et al.*, 2011 ▸). The manganese–calcium and manganese–strontium clusters in solution may likely adopt a structure deviating from the reported crystalline one.

### 3.3. Time and concentration limits for PDFs using an MLM monochromator

The potential benefit of using the MLM over the DCM for PDF experiments is the higher flux and thus potentially increased time resolution, in for example *in situ* experiments, allowing studies of hitherto inaccessible processes. In this section we investigate the effect of the acquisition rate on the reliability of the resulting PDFs.

**Figure 4:**
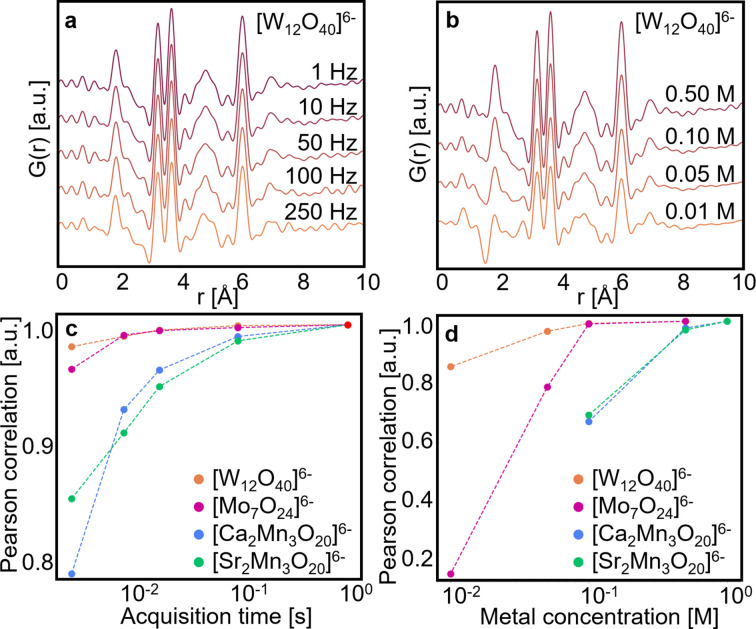
(*a*) Comparison of PDFs obtained from the [W_12_O_40_]^6−^ cluster 0.5 *M* solution with acquisition rates of 250, 100, 50, 10 and 1 Hz. (*b*) Comparison of PDFs obtained from the [W_12_O_40_]^6−^ cluster solution at concentrations of 0.50, 0.10, 0.05 and 0.01 *M*, each measured in 1 s. (*c*) Pearson correlation coefficients between PDFs obtained from 1 *M* cluster solutions with different acquisition times (the 1 s measurement being used as a reference). (*d*) Pearson correlation coefficients between PDFs obtained from cluster solutions with different concentrations (the 1 *M* measurement being used as a reference). All data were collected using the MLM.

Data were collected on the high-concentration samples with acquisition rates of 250, 100, 50, 10 and 1 Hz. As the detector readout time is 1 ms, individual scattering patterns are measured in 0.003, 0.009, 0.019, 0.099 and 0.999 s. The PDFs of the [W_12_O_40_]^6−^ cluster of a single frame obtained at different acquisition rates are shown in Fig. 4 ▸(*a*). Data from the acquisition rate series performed on the three other clusters are shown in Figs. S5(*a*)–S5(*c*) and their respective *F*(*Q*)s are shown in Figs. S6(*a*)–S6(*d*). The scaling factor of the background (*i.e.* DI water and/or 0.5 *M* ammonium persulfate solution) has to be adjusted from one concentration to another (see Table S8). Pearson correlation analysis was carried out in the range 0–10 Å to compare the PDF from the fast measurements with those collected at 1 Hz, as shown in Fig. 4 ▸(*c*). For the [W_12_O_40_]^6−^ and [Mo_7_O_24_]^6−^ clusters in solution, PDFs up to acquisition rates of 250 Hz have a Pearson correlation coefficient above 0.95 with respect to the 1 Hz measurement. This means that, even for a scattering signal measured in only 3 ms, the acquired pattern has a sufficient quality to yield a PDF comparable to those of a 1 s measurement, also for nanostructured samples in solution. The limit of 0.95 is somewhat arbitrary but allows us to quantify ‘high similarity’. For the weaker scattering signal from the two Mn cluster systems the correlation factor of the PDF of the [Ca_2_Mn_3_O_20_]^6−^ and [Sr_2_Mn_3_O_20_]^6−^ clusters falls below 0.95 above 50 Hz. The PDFs of these two clusters with different acquisition rates are shown in Fig. S5(*b*) and S5(*c*). The PDFs of the [Sr_2_Mn_3_O_20_]^6−^ cluster acquired with different acquisition rates are shown in Fig. S5(*c*), where it is clearly seen that the level of noise is high for the PDFs collected at frequencies above 50 Hz compared with the 1 Hz PDF. However, these results are very encouraging and highlight how the high flux provided by an MLM can improve the signal-to-noise ratio in TS experiments on weakly scattering non-crystalline and nanocluster samples and allow studies of processes with time resolution down to tens of milliseconds.

To obtain reasonable signal-to-noise ratios on weakly scattering systems it has often been necessary to increase the concentration of the system under study. It is however well known that concentration can affect the chemical process studied, and concentrations used in, for example, *in situ* PDF studies of chemical synthesis are often much higher (in the 0.5–4 *M* range) than what is used for many material syntheses in the laboratory (Juelsholt *et al.*, 2021 ▸, Bukhtiyarova, 2019 ▸). Leveraging the high flux provided by an MLM, it may be possible to study systems at lower concentrations. A concentration series of 0.50, 0.10, 0.05 and 0.01 *M* was prepared for the [W_12_O_40_]^6−^ and [Mo_7_O_24_]^6−^ samples, and 1.00, 0.50 and 0.10 *M* for the [Ca_2_Mn_3_O_20_]^6−^ and [Sr_2_Mn_3_O_20_]^6−^ samples. The higher concentrations for the Mn-containing samples were chosen due to their weaker scattering power. The PDFs measured for the [W_12_O_40_]^6−^ concentration series at 1 Hz are shown in Fig. 4 ▸(*b*). The scaling factor of the background (*i.e.* DI water and/or 0.5 *M* ammonium persulfate solution) has to be adjusted from one concentration to another (see Table S9). Despite the lower contribution of the metal oxo clusters to the scattering pattern and the increase in the level of noise in their *F*(*Q*) [Figs. S6(*e*)–S6(*h*)], highly similar PDFs are obtained for the four concentrations. Even the PDF from the lowest concentration of 0.01 *M* is quite similar to the 0.5 *M* sample, although it is noisier and the dip around 1.8 Å is more pronounced than for the higher concentrations. Pearson correlations are calculated with respect to the highest-concentration sample in the range 0–10 Å to quantify the similarity of PDFs, as shown in Fig. 4 ▸(*d*). Here it is seen that the correlation factor for [W_12_O_40_]^6−^ is above 0.95 for the higher concentrations but drops below for 0.01 *M*, in line with the PDFs shown in Fig. 4 ▸(*c*). [Mo_7_O_24_]^6−^ concentrations down to 0.10 *M* yield PDFs with correlation above 0.95. PDFs from the other concentration series are shown in Figs. S5(*d*)–S5(*f*). The weaker 0.05 *M* solution of [Mo_7_O_24_]^6−^ [see Fig. S5(*d*)] does show the expected peaks, but the noise is more pronounced and the Pearson correlation coefficient drops to about 0.8. The Pearson correlation coefficient of the 0.01 *M* solution with respect to the 0.5 *M* solution is very low. Comparing the PDFs shown in Fig. S5(*d*) it appears that the structure of the Mo cluster is different in the low-concentration regime compared with the higher concentrations. It does not show order as far in *r* space as the peak at 5.6 Å is not apparent and, moreover, the strong peak observed at 2.8 Å does not relate to any metal–metal distances in the suggested structure. This could indicate that the structure of the cluster is indeed different at a lower concentration, or more likely that at such a low concentration, background subtraction may become imprecise as we reach the detection limit of these clusters by X-rays.

Not unexpectedly, higher concentrations are needed for the weakly scattering manganese clusters to obtain good PDFs. Indeed, only the 0.50 *M* solutions show high correlation with the 1 *M* samples and, for example, the 0.10 *M* concentration yields a correlation coefficient of only 0.65. Note that these measurements are recorded with 1 s exposure time, faster than conventional experiments on these systems (Birgisson *et al.*, 2018 ▸). We did not attempt longer acquisition times on the low-concentration samples.

### 3.4. Radiation-induced effects

**Figure 5:**
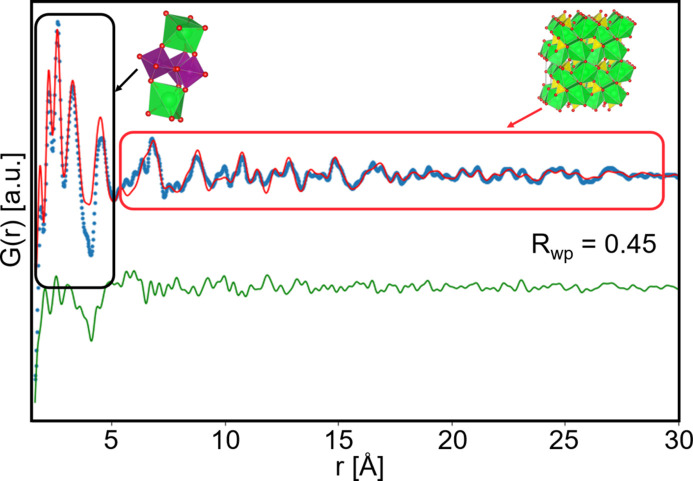
Refinement of the PDF obtained from the [Sr_2_Mn_3_O_20_]^6−^ solution after beam-induced crystallization. Although the PDF still shows the presence of the Sr_2_Mn_3_O_20_ cluster, the long-range order can be described by the SrSO_4_ structure. The experimental PDF is shown in blue, the calculated PDF in red and the difference curve in green.

Radiation-induced effects and radiation damage are ubiquitous at modern synchrotron sources even using highly monochromatic beams (Garman & Weik, 2017 ▸; Lin *et al.*, 2017 ▸; Monico *et al.*, 2020 ▸; Bogdanov *et al.*, 2021 ▸; Thomä & Zobel, 2023 ▸). The very high X-ray flux from the MLM is thus even more likely to induce damage and other effects to the sample. In the case of the manganese clusters in solution, a prolonged exposure of the samples (a few seconds) to the MLM beam led to crystallization, an effect not observed with the DCM beam. When the [Sr_2_Mn_3_O_20_]^6−^ solutions were exposed to the beam for more than a second, precipitation was observed (see Video S1 of the supporting information). In the PDF from the first detector frames, only the [Sr_2_Mn_3_O_20_]^6−^ cluster is observed as shown in Fig. 3 ▸(*e*); however, shortly after, long-range order arises in the PDF as shown in Fig. 5 ▸. This can also be observed from the *F*(*Q*) of the sample when measuring the TS signal for long exposure times, *i.e.* low-acquisition rates [see Figs. S6(*d*) and S6(*h*)]. The resulting long-range order in the PDF can be modeled by the SrSO_4_ (*Pnma*) structure. We imagine that some of the S_2_O_8_^2−^ ions in solution are reduced to SO_4_^2−^ as they oxidize the Mn^2+^ ions during the formation of the manganese clusters (Wang & Li, 2002 ▸). The SO_4_^2−^ ions may react with Sr^2+^ and precipitate as SrSO_4_, triggered by the intense X-ray beam.

In the case of the [W_12_O_40_]^6−^ and [Mo_7_O_24_]^6−^ clusters, no precipitation or change in the PDF was observed over time. However, the solution in the beam path gradually changes from colorless to a bright blue during the exposure as shown in Fig. S7. No structural effect could be observed in the PDF. The color did not disappear but did become weaker due to diffusion in the capillary after X-ray exposure, indicating that this is not caused by a short-lived electronic excitation in W/Mo.

Photon-induced effects should thus be taken into consideration when performing scattering experiments with such high X-ray flux. This is especially true when studying reactions/processes *in situ* as the reaction/process may be perturbed by the X-ray photons.

## 4. Conclusions

Using a high-intensity broad energy bandwidth beam from an MLM monochromator we have shown the feasibility of sub-second TS experiments on nanometre-sized metal oxo clusters in solution. The high photon flux enables the acquisition of data with sufficient quality to retrieve PDFs with a time resolution unachievable with a traditional DCM. The broad energy bandwidth leads to damping of the PDF signal and broadening of the PDF peaks. This in turn limits the usable *r* range compared with the DCM. In practice, however, this difference is minimal for the geometry used in this particular experiment, which reduces the advantage of the DCM. With a 1 mm capillary sample, it is possible to obtain PDFs up to ∼60 Å, and thus yield sufficient real space resolution to study disordered systems such as metal oxo clusters in solution and many other materials. Using higher energies and longer sample-to-detector distances the difference in usable *r* range is expected to be larger.

The high flux provided by the MLM allows time-resolved studies with a time resolution well below a second for a wide range of small nanometre-sized systems. Depending on the scattering power and concentration of the samples, high-quality PDFs can be obtained in 100 ms and in some cases as little as 3 ms. The high flux can also be leveraged to study more dilute systems, allowing greater flexibility in the design of the reaction conditions.

The work presented here shows the feasibility and the potential of using an MLM monochromator for *in situ* studies of very small nanoclusters and nanoparticles at much greater time resolution than previously possible.

## Supplementary Material

Video S1. DOI: 10.1107/S1600576723004016/vb5050sup1.mp4

Tables of refined parameters, PDF of Si using DCM and MLM, Correlation matrices from refinements, beam spectrum etc. DOI: 10.1107/S1600576723004016/vb5050sup2.pdf
