# Supplementary material for: Sub-second pair distribution function using a broad bandwidth monochromator
Source: J Appl Crystallogr. 2023 May 31;56(Pt 3):825–33. doi: 10.1107/S1600576723004016 (PMC10241043; doi:10.1107/S1600576723004016)
Supplement: Supplementary file 2 [file j-56-00825-sup2.pdf]

JOURNAL OF APPLIED CRYSTALLOGRAPHY

Volume 56 (2023)

Supporting information for article:

Sub-second pair distribution function using a broad bandwidth monochromator

Nicolas P. L. Magnard, Daniel R. Sørensen, Innokenty Kantor, Kirsten M. Ø. Jensen and Mads R. V. Jørgensen

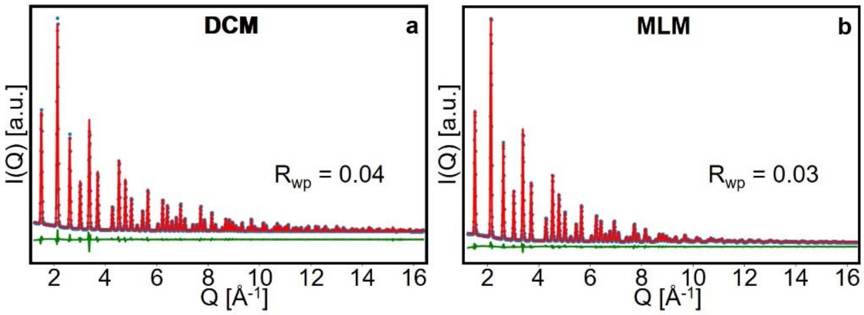

Figure S1: Rietveld refinement against the LaB_6 standard measured using the a) DCM, and b) MLM

Parameter	DCM (PXRD)	DCM (PDF)	MLM (PXRD)	MLM (PDF)
U [a.u.]	0.064(5)	-	-0.271(2)	-
V [a.u.]	0.0257(3)	-	-0.0139(4)	-
W [a.u.]	-0.0272(3)	-	-0.0197(3)	-
Y [a.u.]	0.0724(5)	-	0.0425(8)	-
Q_{damp} [Å^{-1}]	-	0.0150(1)	-	0.0193(1)
Q_{broad} [Å^{-1}]	-	0.0001(3)	-	0.0331(5)
Delta2 [Å^{-2}]	-	1.74(5)	-	1.13(2)
Scale [a.u.]	0.0000809(1)	0.439(1)	0.00159(3)	0.556(1)
a (LaB_6 $Pm\bar{3}m$) [Å]	4.1543(4)	4.1545(1)	4.1554(3)	4.1554(1)
x_{Boron}	0.1998(4)	0.1991(3)	0.2013(4)	0.1998(3)
B_{iso}(La) [Å^{-2}]	0.453(6)	0.330(3)	0.5393(7)	0.463(4)
B_{iso}(B) [Å^{-2}]	0.418(3)	0.99(1)	0.3529(3)	1.14(1)

Table S1: Results of the PXRD and PDF refinement against the LaB_6 data obtained from the DCM and MLM TS measurements. Refined values are shown along with their estimated standard deviation in parenthesis.

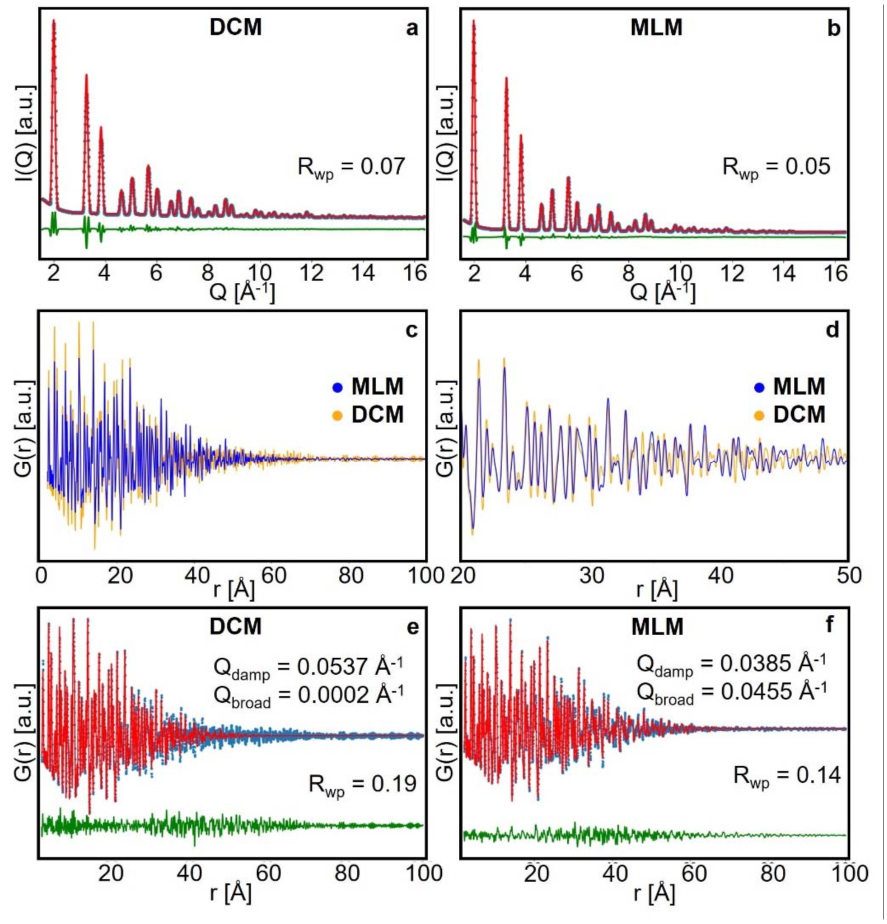

Figure S2: a) PXRD refinement of the Si structure ($Fd\bar{3}m$) against the Si standard data in a ⌀1 mm glass capillary using the DCM. b) PXRD refinement of the Si structure ($Fd\bar{3}m$) against the Si standard data in a ⌀1 mm glass capillary using the MLM c) Comparison of the PDF obtained using the DCM (orange curve) and the MLM (blue curve). d) Zoom in the [20-50] Å range. e) Refinement of the Si structure ($Fd\bar{3}m$) on the DCM PDF where Q_{damp} and Q_{broad} are refined. f) Refinement of the Si structure ($Fd\bar{3}m$) on the MLM PDF where Q_{damp} and Q_{broad} are refined. (Experimental data are in blue dots, model shown in red curve and difference in green in a, b, e and f)

Parameter	DCM (PXRD)	DCM (PDF)	MLM (PXRD)	MLM (PDF)
U [a.u.]	0.797(3)	-	0.00729(7)	-
V [a.u.]	-0.143(6)	-	-0.0072(3)	-
W [a.u.]	-0.1535(3)	-	-0.118(3)	-
Y [a.u.]	0.0001(7)	-	0.0001(4)	-
Q_{damp} [Å^{-1}]	-	0.0389(2)	-	0.0385(2)
Q_{broad} [Å^{-1}]	-	0.0001(2)	-	0.0486(1)
Delta2 [Å^{-2}]	-	5.2(4)	-	2.9(1)

Scale [a.u.]	0.00096(6)	0.2353(1)	0.00954(3)	0.2622(8)
a (Si $Fd\bar{3}m$) [Å]	5.429(2)	5.4279(8)	5.442(1)	5.43791(7)
B_{iso}(Si) [$Å^{-2}$]	0.424(2)	0.324(8)	0.494(2)	0.316(5)

Table S2:

Table S2: Results of the PXRD and PDF refinement against the Si data obtained from the DCM and MLM TS measurements. Refined values are shown along with their estimated standard deviation in parenthesis.

Parameter	DCM	MLM
$Gauss_{damp}$ [$Å^{-1}$]	0.0311(1)	0.0420(1)
$Gauss_{broad}$ [$Å^{-1}$]	0.0001(4)	0.0038(1)
Lor_{damp} [$Å^{-1}$]	0.0063(1)	0.0031(1)
Lor_{broad} [$Å^{-1}$]	0.0001(2)	0.0009(1)
Delta1 [$Å^{-2}$]	0.001(5)	0.15(2)
Scale [a.u.]	0.472(1)	0.568(1)
a (LaB_6 $Pm\bar{3}m$) [Å]	4.1545(1)	4.1555(1)
x_{Boron}	0.1990(3)	0.1994(3)
B_{iso}(La) [$Å^{-2}$]	0.328(3)	0.486(5)
B_{iso}(B) [$Å^{-2}$]	0.98(2)	1.34(2)

Table S3: Results of the refinement of the LaB_6 PDF obtained from the DCM and MLM TS measurements using a Voigt profile. Refined values are shown along with their estimated standard deviation in parenthesis

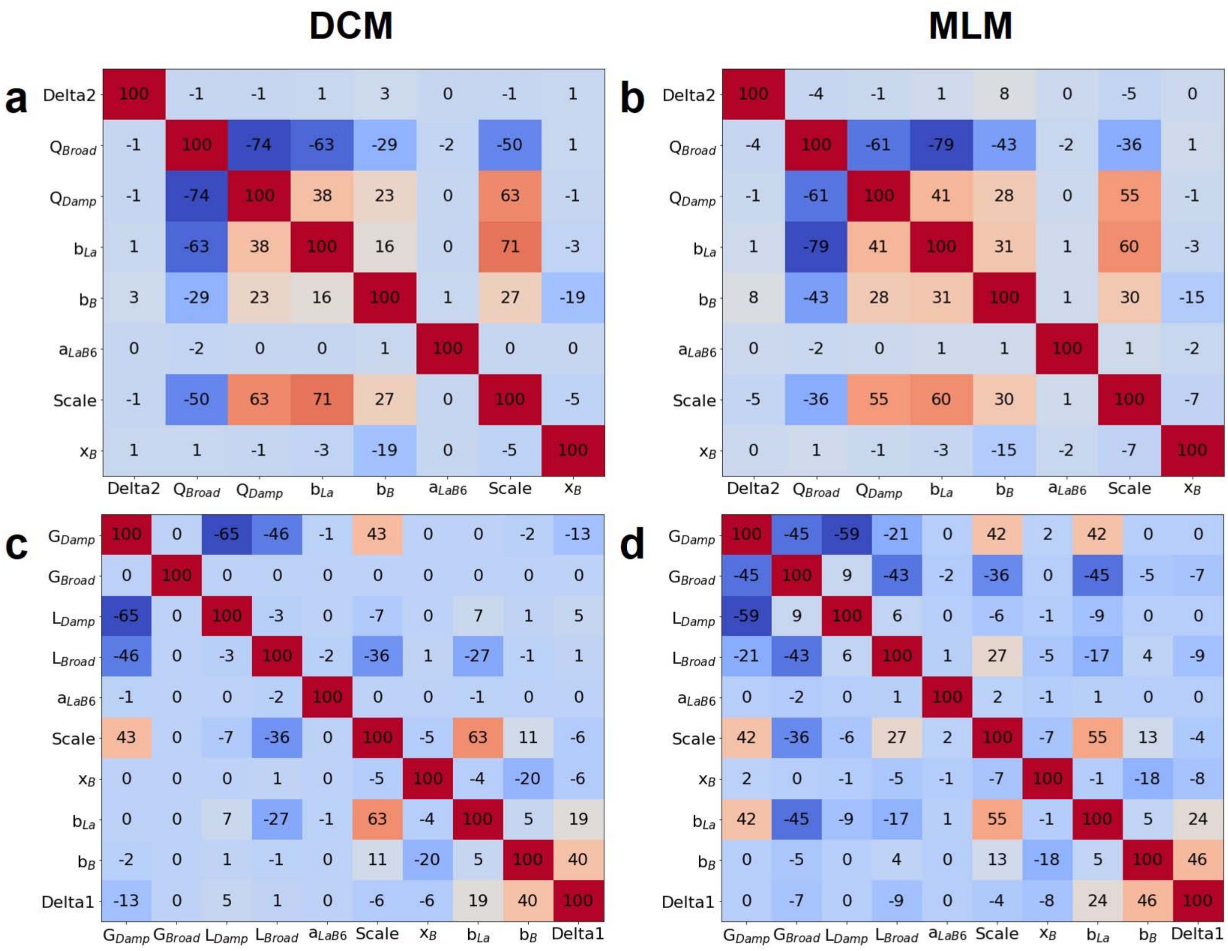

Figure S3: a) Correlation matrix of refined parameters during the PDF refinement of the DCM LaB_6 data using a "regular" model. The same correlation matrix for the refinement of the MLM data in b). In c) and d) are shown the correlation matrixes of the refinement of the DCM and MLM data, respectively, using a Voigt peak profile.

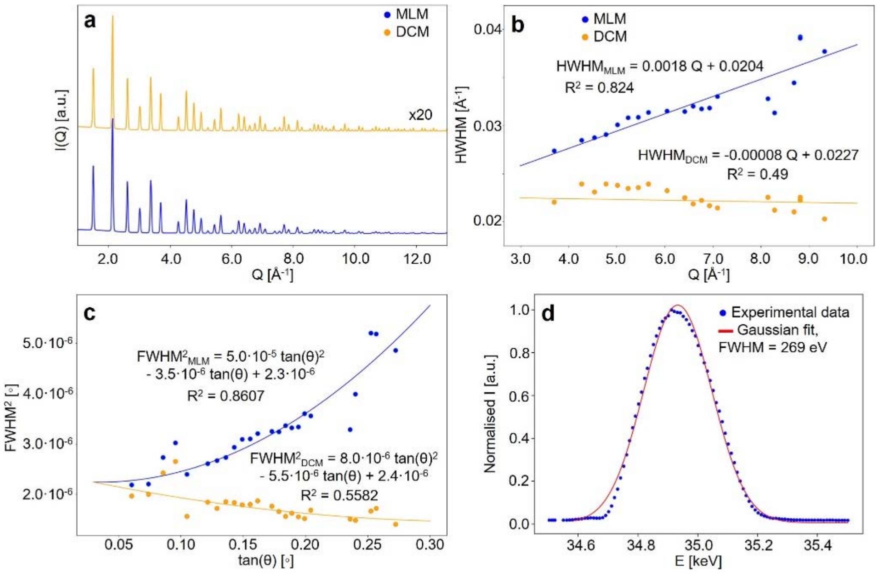

Figure S4: a) TS patterns of a LaB_6 standard measured using the MLM (blue) and DCM (orange). b) Half-Width at Half-Maximum of Bragg peaks of the LaB_6 TS pattern collected using MLM (blue) and DCM (orange) plotted as a function of the momentum transfer, Q. In order to emphasize the difference in Q-dependence of the peaks broadening between the two measurement, a linear regression is plotted over each dataset. c) Instrumental Resolution Function (IRF) of the DCM and MLM, measured on the LaB6 scattering patterns. d) Energy distribution of the X-ray beam when using the MLM. A Gaussian profile (red curve) could be fitted to the experimental data (blue dots) and the FWHM of the model could be extracted.

Parameter	DCM	MLM
Delta2 [Å^{-2}]	3.47(1)	3.468(6)
Scale [a.u.]	0.302(4)	0.29(3)
a [Å]	18.77(1)	18.77(4)
b [Å]	18.94(1)	18.94(3)
c [Å]	25.86(1)	25.840(3)
α [°]	91.04(8)	90.89(2)
β [°]	89.41(7)	89.29(4)
γ [°]	120.50(3)	120.50(8)
B_{iso}(W) [Å^{-2}]	0.0026(3)	0.0078(3)
B_{iso}(O) [Å^{-2}]	0.028(6)	0.022(4)

Atom	X	Y	Z
W1	0.581(6)	0.436(5)	0.315(3)
W2	0.419(5)	0.563(2)	0.314(1)
W3	0.44(1)	0.581(1)	0.519(2)
W4	0.56(1)	0.417(7)	0.516(3)
W5	0.635(5)	0.627(8)	0.319(1)
W6	0.362(8)	0.372(2)	0.321(1)
W7	0.63(1)	0.636(5)	0.513(2)
W8	0.371(9)	0.363(4)	0.512(3)
W9	0.702(5)	0.546(8)	0.411(3)
W10	0.298(3)	0.449(5)	0.411(2)
W11	0.546(8)	0.703(4)	0.420(2)
W12	0.450(9)	0.300(4)	0.422(3)
O1	0.5993	0.4972	0.3493
O2	0.3913	0.4990	0.3460
O3	0.4982	0.6063	0.4833
O4	0.4985	0.3972	0.4833
O5	0.6008	0.3826	0.2758
O6	0.3941	0.6125	0.2762
O7	0.3833	0.6026	0.5581
O8	0.6213	0.3923	0.5653
O9	0.7110	0.7238	0.2829
O10	0.3084	0.2952	0.2827

O11	0.7215	0.7169	0.5500
O12	0.2704	0.3042	0.5486
O13	0.7757	0.5746	0.3940
O14	0.2227	0.4272	0.4008
O15	0.5751	0.7821	0.4171
O16	0.4228	0.2055	0.4087
O17	0.6597	0.5335	0.2809
O18	0.3699	0.4889	0.2961
O19	0.5372	0.6666	0.5463
O20	0.4608	0.3340	0.5446
O21	0.6824	0.4681	0.3494
O22	0.3012	0.5305	0.3511
O23	0.4631	0.6947	0.4827
O24	0.5271	0.3143	0.4830
O25	0.4717	0.4028	0.2941
O26	0.5104	0.5895	0.2962
O27	0.3997	0.4709	0.5374
O28	0.5854	0.5223	0.5393
O29	0.6144	0.6847	0.3719
O30	0.3865	0.3024	0.3662
O31	0.6830	0.6197	0.4558
O32	0.3018	0.3883	0.4548
O33	0.5356	0.3427	0.3715
O34	0.4595	0.6545	0.3708
O35	0.3486	0.5382	0.4591
O36	0.6525	0.4556	0.4569
O37	0.7357	0.6474	0.3507
O38	0.2681	0.3702	0.3511
O39	0.6487	0.7452	0.4733
O40	0.3504	0.2606	0.4827

Table S4: Results of the refinement of the $[W_{12}O_{40}]^{6-}$ *cluster solution PDF obtained from the DCM and MLM TS measurements.*

Parameter	DCM	MLM
Delta2 [Å^{-2}]	2.78(1)	2.78(1)
Scale [a.u.]	0.68(5)	0.68(3)
a [Å]	16.71(8)	16.73(1)
b [Å]	35.96(2)	35.970(5)
c [Å]	21.27(3)	21.270(4)
α [°]	89.64(1)	89.67(3)
β [°]	115.40(1)	115.40(2)
γ [°]	89.57(2)	89.54(5)
B_{iso}(Mo) [Å^{-2}]	0.0037(1)	0.0037(1)
B_{iso}(O) [Å^{-2}]	0.006(6)	0.007(5)

Atom	X	Y	Z
Mo1	0.220(2)	0.393(2)	0.4311(6)
Mo2	0.16(2)	0.448(2)	0.297(2)
Mo3	0.09(1)	0.3037(6)	0.291(2)
Mo4	0.018(8)	0.358(1)	0.161(2)
Mo5	0.28(2)	0.308(2)	0.427(4)
Mo6	0.155(9)	0.425(2)	0.148(4)
Mo7	0.24(1)	0.365(1)	0.287(3)
O1	0.3264	0.4276	0.5020
O2	0.2813	0.4780	0.3605
O3	0.0864	0.2742	0.2147
O4	0.1581	0.3760	0.4993
O5	0.0584	0.4820	0.2128
O6	0.0479	0.3098	0.3521
O7	0.2161	0.2912	0.4922
O8	0.0761	0.4532	0.0701
O9	0.3790	0.2767	0.4400
O10	0.2268	0.4386	0.1094
O11	0.3206	0.3559	0.4524
O12	0.2208	0.4507	0.2330
O13	0.2009	0.2871	0.3461
O14	0.0947	0.3810	0.1051
O15	0.3094	0.3267	0.3608

O16	0.2813	0.4066	0.2372
O17	0.1230	0.4110	0.3516
O18	0.0255	0.3473	0.2462
O19	0.2602	0.4074	0.3483
O20	0.1527	0.3289	0.2496
O21	0.1843	0.3499	0.3551
O22	0.1357	0.4013	0.2169
O23	-0.0010	0.3210	0.1000
O24	0.0633	0.3936	0.1221

Table S5: Results of the refinement of the $[Mo_7O_{24}]^{6-}$ *cluster solution PDF obtained from the DCM and MLM TS measurements.*

Parameter	DCM	MLM
Delta2 [Å^{-2}]	2.7(6)	2.6(3)
Scale [a.u.]	0.32(7)	0.23(5)
a [Å]	45.62(5)	45.90(5)
b [Å]	42.57(4)	42.29(5)
c [Å]	21.17(4)	21.3(6)
α [°]	86.8(1)	87.2(2)
β [°]	88.4(1)	87.9(1)
γ [°]	90.7(1)	87.6(1)
B_{iso}(Mn) [Å^{-2}]	0.0130(2)	0.0084(2)
B_{iso}(Ca) [Å^{-2}]	0.010(8)	0.005(3)
B_{iso}(O) [Å^{-2}]	0.023(6)	0.025(6)

Atom	X	Y	Z
Mn1	0.58(1)	0.531(9)	0.07(3)
Mn2	0.637(3)	0.573(8)	0.14(2)
Mn3	0.570(5)	0.581(7)	0.16(4)
Ca1	0.59(2)	0.607(7)	0.02(3)
Ca2	0.61(1)	0.516(8)	0.21(4)
O1	0.62121	0.45938	0.22618
O2	0.58123	0.49084	0.13190
O3	0.57119	0.55777	0.02176
O4	0.63659	0.49662	0.29630
O5	0.65315	0.53612	0.22157
O6	0.62849	0.59742	0.08520
O7	0.62202	0.64996	0.04962
O8	0.56663	0.49292	0.27762
O9	0.57026	0.54719	0.22546
O10	0.56347	0.60297	0.11104
O11	0.56181	0.64992	0.04714
O12	0.60398	0.55089	0.13090
O13	0.54609	0.50069	0.00663
O14	0.68143	0.58660	0.18801
O15	0.53726	0.59577	0.22013
O16	0.65531	0.52825	0.10787

O17	0.60919	0.59286	0.21763
O18	0.53775	0.54157	0.13381
O19	0.55729	0.59875	-0.05635
O20	0.62564	0.59962	-0.06615

Table S6: Results of the refinement of the $[Ca_2Mn_3O_{20}]^{6-}$ *cluster solution PDF obtained from the DCM and MLM TS measurements.*

Parameter	DCM	MLM
Delta2 [Å^{-2}]	2.6(1)	2.9(3)
Scale [a.u.]	0.15(4)	0.23(1)
a [Å]	42.8(5)	42.9(4)
b [Å]	42.4(4)	42.9(2)
c [Å]	22.9(3)	23.04(9)
α [°]	91.8(1)	90.6(6)
β [°]	90.5(2)	88.8(8)
γ [°]	89.8(2)	89.3(6)
B_{iso}(Mn) [Å^{-2}]	0.005(1)	0.0009(2)
B_{iso}(Sr) [Å^{-2}]	0.002(4)	0.003(3)
B_{iso}(O) [Å^{-2}]	0.02(3)	0.21(2)

Atom	X	Y	Z
Mn1	0.59(4)	0.524(5)	0.075(2)
Mn2	0.64(6)	0.56(7)	0.161(2)
Mn3	0.57(4)	0.57(4)	0.186(2)
Sr1	0.59(6)	0.62(4)	0.024(2)
Sr2	0.60(5)	0.50(3)	0.213(2)
O1	0.60806	0.43770	0.21152
O2	0.58400	0.49419	0.12303
O3	0.57254	0.56027	0.01399
O4	0.64018	0.48634	0.30729
O5	0.64585	0.54443	0.20702
O6	0.63381	0.60051	0.08222
O7	0.62513	0.67249	0.01130
O8	0.56482	0.47287	0.28793
O9	0.56806	0.54825	0.23348
O10	0.56574	0.60770	0.11211
O11	0.55980	0.67397	0.02736
O12	0.59938	0.55475	0.12635
O13	0.55465	0.50252	0.01545
O14	0.67780	0.59241	0.15349
O15	0.53818	0.60258	0.21135
O16	0.66204	0.53758	0.08012

O17	0.60979	0.60036	0.21018
O18	0.53629	0.54594	0.11968
O19	0.56285	0.60364	0.42152
O20	0.62326	0.59724	0.42596

Table S7: Results of the refinement of the $[Sr_2Mn_3O_{20}]^{6-}$ *cluster solution PDF obtained from the DCM and MLM TS measurements.*

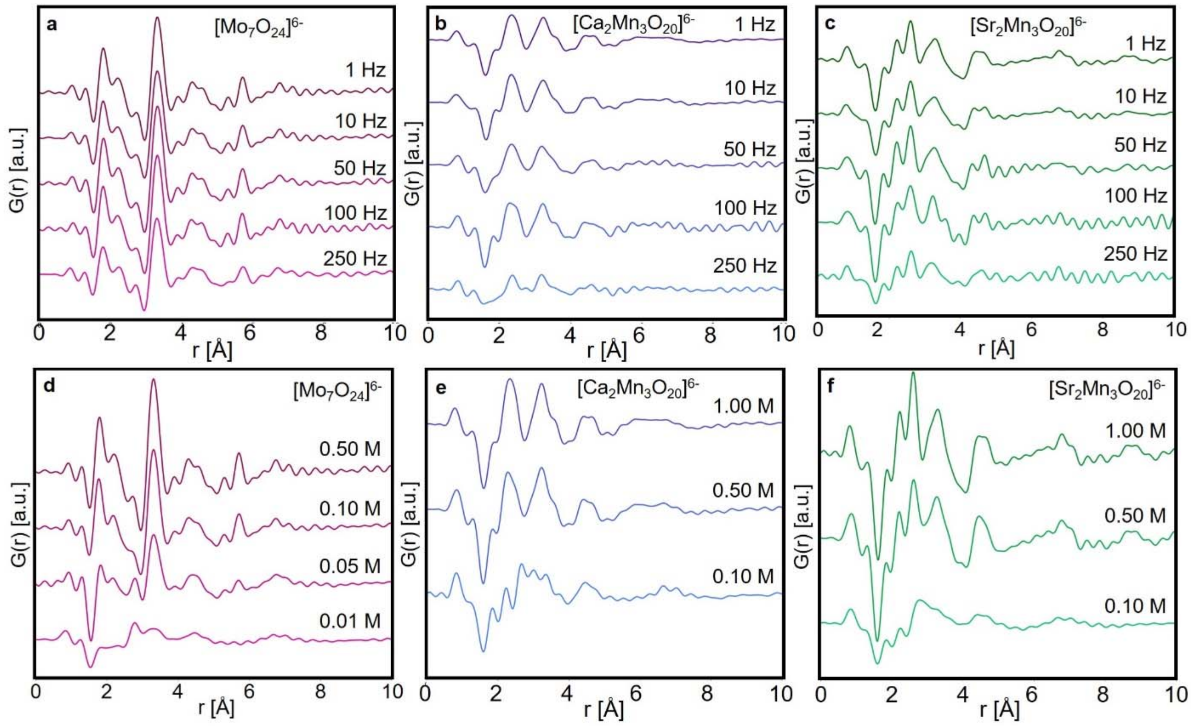

Figure S5: a) Comparison of PDFs measured on the $[Mo_7O_{24}]^{6-}$, b) $[Ca_2Mn_3O_{20}]^{6-}$ and c) $[Sr_2Mn_3O_{20}]^{6-}$ clusters 0.50 M solution with acquisition rate of 250 Hz, 100 Hz, 50 Hz, 10 Hz and 1 Hz. d) Comparison of PDFs measured on the $[Mo_7O_{24}]^{6-}$, e) $[Ca_2Mn_3O_{20}]^{6-}$ and f) $[Sr_2Mn_3O_{20}]^{6-}$ clusters solutions with concentrations of 0.50, 0.10, 0.05 and 0.01 M for the $[Mo_7O_{24}]^{6-}$, and 1.00, 0.50 and 0.10 M for $[Ca_2Mn_3O_{20}]^{6-}$ and $[Sr_2Mn_3O_{20}]^{6-}$.

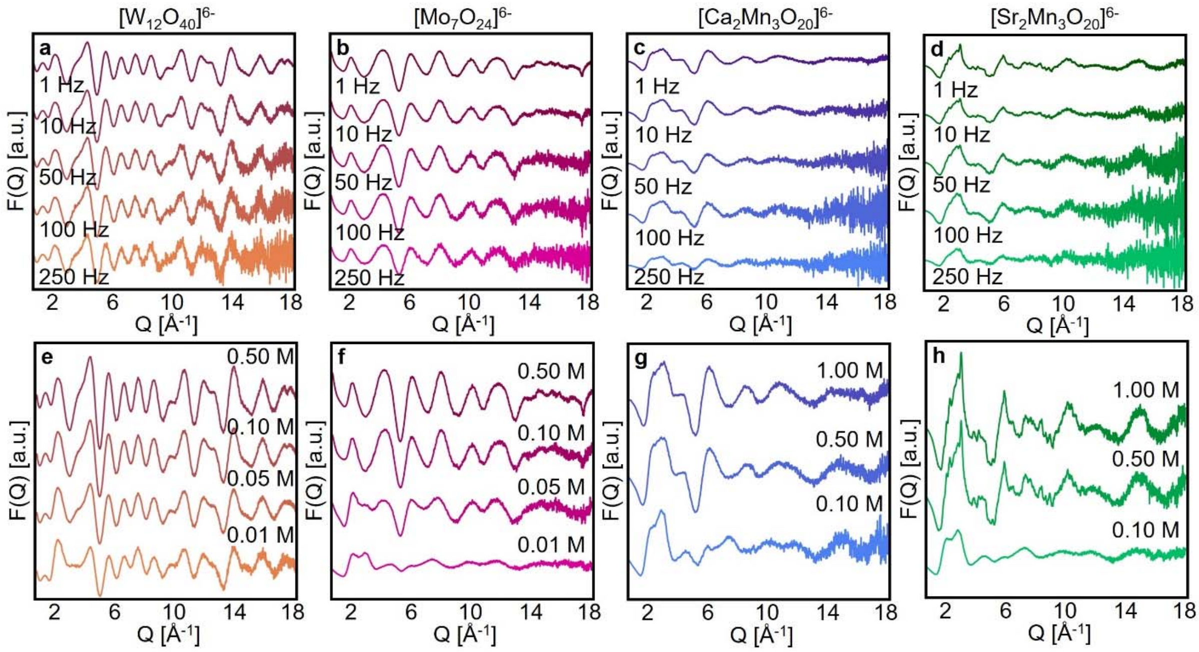

Figure S6: a) Comparison of F(Q) measured on the $[W_{12}O_{40}]^{6-}$, b) $[Mo_7O_{24}]^{6-}$, c) $[Ca_2Mn_3O_{20}]^{6-}$ and d) $[Sr_2Mn_3O_{20}]^{6-}$ clusters 0.50 M solution with acquisition rate of 250 Hz, 100 Hz, 50 Hz, 10 Hz and 1 Hz. e) Comparison of F(Q) measured on the $[W_{12}O_{40}]^{6-}$, f) $[Mo_7O_{24}]^{6-}$, g) $[Ca_2Mn_3O_{20}]^{6-}$ and h) $[Sr_2Mn_3O_{20}]^{6-}$ clusters solutions with concentrations of 0.50, 0.10, 0.05 and 0.01 M for $[W_{12}O_{40}]^{6-}$ and $[Mo_7O_{24}]^{6-}$, and 1.00, 0.50 and 0.10 M for $[Ca_2Mn_3O_{20}]^{6-}$ and $[Sr_2Mn_3O_{20}]^{6-}$.

Acquisition rate	$[W_{12}O_{40}]^{6-}$	$[Mo_7O_{24}]^{6-}$	$[Ca_2Mn_3O_{20}]^{6-}$	$[Sr_2Mn_3O_{20}]^{6-}$
1 Hz	0.06348	0.74528	1.15384	1.07948
10 Hz	0.00638	0.06796	0.11464	0.10418
50 Hz	0.00118	0.01444	0.02132	0.02098
100 Hz	0.00052	0.00654	0.01054	0.01004
250 Hz	0.00016	0.00194	0.00296	0.00296

Table S8: Scaling factor of the background used during data reduction (DI water for for $[W_{12}O_{40}]^{6-}$ and $[Mo_7O_{24}]^{6-}$, and a 0.5 M ammonium persulfate solution for $[Ca_2Mn_3O_{20}]^{6-}$ and $[Sr_2Mn_3O_{20}]^{6-}$) of the total scattering patterns measured at various acquisition rates, namely, 1, 10, 50, 100 and 250 Hz.

Concentration	$[W_{12}O_{40}]^{6-}$	$[Mo_7O_{24}]^{6-}$	$[Ca_2Mn_3O_{20}]^{6-}$	$[Sr_2Mn_3O_{20}]^{6-}$
1.00 M	-	-	1.15384	1.07948
0.50 M	0.06348	0.74528	1.43174	1.45644
0.10 M	0.34992	0.97588	1.19596	1.41874

0.05 M	0.63322	0.93002	-	-
0.01 M	1.03172	1.1515	-	-

Table S9: Scaling factor of the background used during data reduction (DI water for $[W_{12}O_{40}]^{6-}$ and $[Mo_7O_{24}]^{6-}$, and a 0.5 M ammonium persulfate solution for $[Ca_2Mn_3O_{20}]^{6-}$ and $[Sr_2Mn_3O_{20}]^{6-}$) of the total scattering patterns measured at various concentrations, namely, 0.50, 0.10, 0.05 and 0.01 M for $[W_{12}O_{40}]^{6-}$ and $[Mo_7O_{24}]^{6-}$, and 1.00, 0.50 and 0.10 M for $[Ca_2Mn_3O_{20}]^{6-}$ and $[Sr_2Mn_3O_{20}]^{6-}$.

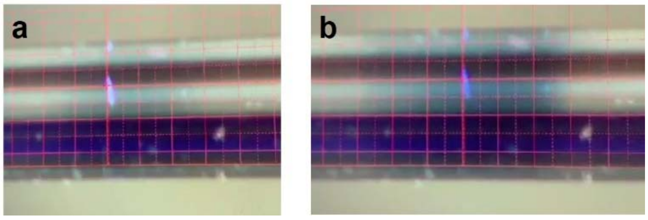

Figure S7: Picture of a capillary containing the $[W_{12}O_{40}]^{6-}$ *cluster solution after 1 s (a) and 10 s (b) of exposure to the MLM X-ray beam. The blue area on the screen if the one where the X-rays impinge on the sample.*
